# Viva lavidaviruses! Five features of virophages that parasitize giant DNA viruses

**DOI:** 10.1371/journal.ppat.1007592

**Published:** 2019-03-21

**Authors:** Sarah Duponchel, Matthias G. Fischer

**Affiliations:** Department of Biomolecular Mechanisms, Max Planck Institute for Medical Research, Heidelberg, Germany; University of Florida, UNITED STATES

## Introduction

Viruses are obligate intracellular parasites and thus cannot replicate outside a living cell. Whereas a susceptible and permissive host cell is sufficient for most viruses to produce infectious progeny, some viruses such as satellite viruses require two different biological entities for their replication. In contrast to most satellite viruses with small RNA-based genomes, the lavidaviruses (or virophages) that replicate in eukaryotic microbes (protists) are comparable to adenoviruses in terms of particle size and genetic complexity. Lavidaviruses only replicate during a co-infection with a giant DNA virus (GV; see, e.g., [1]), and the first described virophage, Sputnik, was serendipitously discovered during a search for new GVs [2].

## What are virophages?

**Fig 1:**
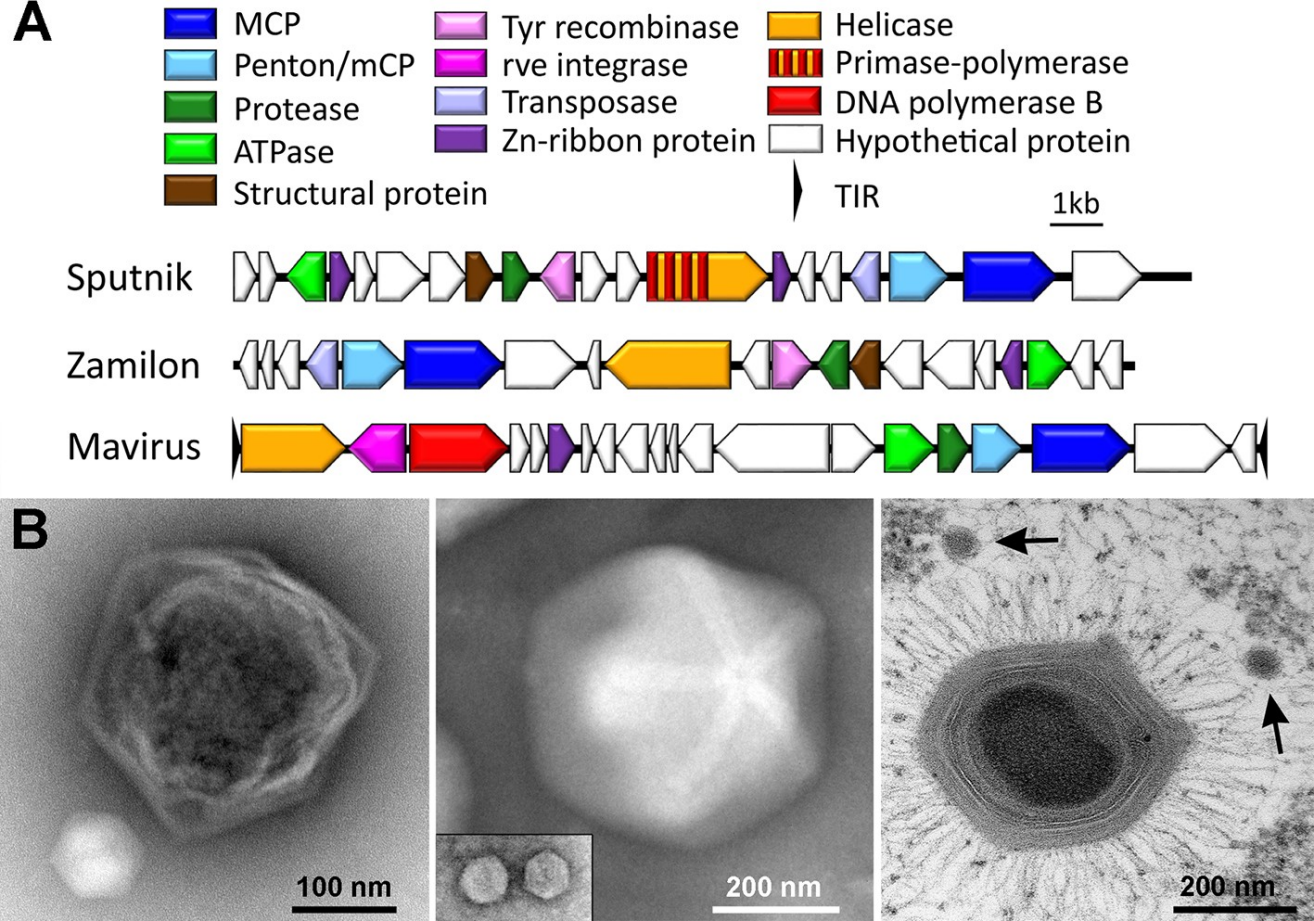
Genome organization and capsid shape of cultured virophages. (A) Genome representation of the virophages Sputnik, Zamilon, and mavirus. Homologous genes are colored identically. (B) Electron microscopy images depicting capsids of giant viruses and their associated virophages. (Left) CroV (dark) and mavirus (light); negative stain EM courtesy of U. Mersdorf, MPI for Medical Research, Germany. (Middle) Megavirus vitis (with a visible stargate structure) and Zamilon vitis (inset); negative stain EM courtesy of C. Abergel, Aix-Marseille Université, France. (Right) Acanthamoeba polyphaga mimivirus with two Sputnik virus particles (arrows); thin-section EM courtesy of J.Y. Bou Khalil and B. La Scola, IHU Mediterranée Infection, France. Note that all three virophages have similar capsid sizes but are shown here at different magnifications. EM, electron microscopy; CroV, Cafeteria roenbergensis virus; TIR, terminal inverted repeat.

Viruses of the family *Lavidaviridae* (large-virus dependent or associated viruses), commonly known as virophages, have double-stranded 17 to 30 kb long DNA genomes of linear or circular topology [2–4]. Their nonenveloped capsids are 50 to 75 nm in diameter and have icosahedral symmetry with a triangulation number of 27 [5]. The family currently comprises two genera, *Mavirus* and *Sputnikvirus* [3], with one and six isolates, respectively. Notwithstanding, as more virophages are isolated or identified in metagenomes, the family is likely to expand [6,7]. Lavidaviruses infect protists but replicate only in the presence of a suitable GV co-infecting the same host cell. During a co-infection, virophages require energy, ribosomes, and metabolites from the cell, but they do not cause cytopathic effects in the cellular host alone. Based on their ability to inhibit the replication of GVs during a co-infection, virophages are primarily parasites of GVs and not of the host cell [2,8]. The term “virophage” reflects this status of a “virus of a virus” [2]. The virophages Sputnik and mavirus replicate at the expense of their GVs *Acanthamoeba polyphaga* mimivirus (APMV) and *Cafeteria roenbergensis* virus (CroV), respectively [2,4] (Fig 1). Another Sputnik-related virophage called Zamilon also requires co-infection of the amoebal host with a GV but does not affect replication of the latter [9]. Dependence on GVs thus appears to be a common feature of virophages, whereas their pathogenic effect on GVs varies.

## How do virophages replicate?

**Fig 2:**
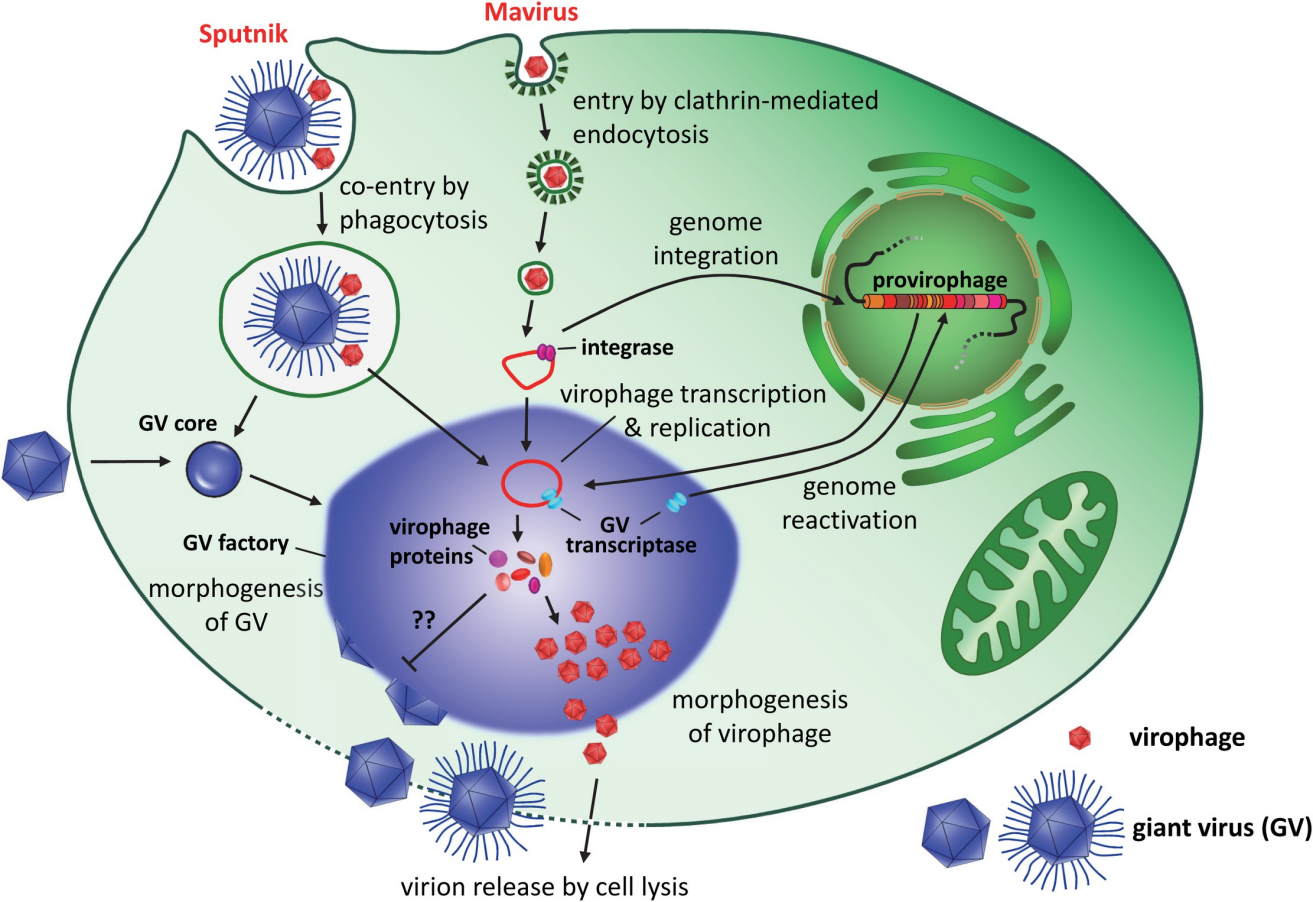
Proposed GV and virophage infection cycle in a eukaryotic host cell. Some GV capsids (e.g., mimivirus) are covered in fibers that allow co-entry of virophages (e.g., Sputnik) by phagocytosis. Other virophages such as mavirus enter cells by receptor-mediated endocytosis. After opening of the GV capsid and fusion of the internal GV membrane with the phagosomal or cytoplasmic membrane, the GV core is released into the cytoplasm and develops into the viral factory. The virophage genome is targeted to the factory, where the GV-encoded transcriptase complex activates virophage genes during the late phase of GV infection. Virophage genome replication is catalyzed by virophage-encoded DNA polymerases and helicases, and virophage particles are assembled within or near the GV factory and are released upon cell lysis. Virophage replication can inhibit GV production. Alternatively, the mavirus genome is able to integrate into the nuclear host genome independently of a GV. The otherwise transcriptionally silent provirophage genes can be activated during infection with a compatible GV, leading to the production of virophage particles in the GV factory. GV, giant virus.

The replication cycles of virophages are still poorly understood, but certain key features are starting to emerge (Fig 2). Different virophages use different pathways for cell entry: whereas mavirus particles enter the host cell independently of CroV by receptor-mediated endocytosis [4], Sputnik virions attach to the glycosylated protein fibers that coat the mimivirus capsid and are thought to enter the cell as a composite by phagocytosis [10]. Packaging of Sputnik particles within mimivirus capsids has also been described [2]; however, the relevance of these nested structures for entry and infection are not known. After entry, the virophage genome is released from the endosome or phagosome and locates to the cytoplasmic GV factory, where virophage replication takes place. The GV factory is a coherent, nonmembrane-bound structure that is composed of hundreds of GV-encoded proteins that act in concert to assemble new GV particles [11]. Among these viral proteins is a presumably complete set of transcription enzymes that enable mimiviruses to replicate entirely in the host cytoplasm, a feature they share with poxviruses [12]. Two temporal gene classes, corresponding to early and late gene expression, have been identified in mimiviruses along with their regulatory sequences [13,14]. APMV and CroV share the late gene promoter motifs and transcription termination signals with their associated virophages, Sputnik and mavirus, suggesting that virophage gene expression is initiated by GV-encoded transcription factors during the late phase of GV infection [4,15]. Genome replication is probably catalyzed by virophage-encoded DNA polymerases and helicases. Capsid assembly and maturation requires at least four different viral gene products: the major capsid protein (MCP), the penton protein (also called minor capsid protein [mCP]), a predicted DNA-pumping ATPase, and a cysteine protease (Fig 1A). The protease cleaves the C-terminal part of the MCP, which is likely required for virion maturation [16]. Virophage particles are released upon cell lysis [2,17].

## What makes virophages evolutionarily successful?

The genomes of virophages are modular in nature. The morphogenesis gene module (MCP, penton, PRO, ATPase) is found in all virophage genomes, whereas the replication module, consisting of DNA polymerase, helicase, and integrase, is not conserved and is subject to nonhomologous gene replacement [18]. At least 50% of a typical virophage genome is composed of genes of unknown ancestry and function. One of the evolutionary roots of virophages may lie with a tectivirus-like ancestor that recombined with eukaryotic transposons [19]; therefore, frequent gene exchange and recombination with various mobile genetic elements appear to be driving forces of virophage evolution [18]. The integrase genes are of particular importance because they allow virophages to permanently associate with other genomes as “provirophages.” Sputnik and Zamilon encode a predicted tyrosine recombinase and integration of Sputnik in the genome of a mimivirus strain has been reported [20]. In contrast, mavirus-type virophages and provirophages in the microalga *Bigelowiella natans* [21] encode a retroviral integrase. In mavirus, the integrase is packaged in the capsid [16], and the viral genome integrates efficiently into the nuclear genome of its flagellate host *Cafeteria roenbergensis*, where the resulting provirophage genome is transcriptionally silent [8]. Infection of a mavirus-bearing cell with CroV can trigger the activation of mavirus genes and the production of mavirus particles (Fig 2). The dual lifestyle of integrating virophages may help them to persist during times in which compatible GVs are scarce. Endogenous mavirus elements could enhance survival of GV-infected host populations, and the mutualistic relationship with *C*. *roenbergensis* may have favored their dissemination and diversification [8]. Strong evidence for the long-standing association with eukaryotic genomes stems from the close genetic relationship between mavirus-type virophages and the endogenous Maverick/Polinton elements. These mobile genetic elements occur in various eukaryotic genomes and replicate either via transposition or through the help of a secondary virus [4,18,22,23]. Therefore, their high genomic mobility, close connection to eukaryotes, and frequent interaction with other mobile genetic elements may have contributed to the evolutionary success of virophages.

## How do virophages influence the ecosystem?

Co-infection of Sputnik or mavirus with their respective GV leads to decreased GV production and better survival of host-cell populations [2,8]. Although the molecular mechanisms underlying virophage parasitism and the extent to which virophages inhibit GVs in natural ecosystems are unknown, modeling approaches suggest that lavidaviruses influence the population dynamics of GVs and protists alike, with consequences for nutrient cycling and primary production [24,25]. Lavidaviruses are associated with different protist lineages, including heterotrophic Stramenopiles and Amoebozoa, but most likely also photosynthetic microeukaryotes [6,26]. Virophage metagenomes have been assembled from different habitat types, including oceans, lakes, soil, and animal digestive tracts [7]. In addition to immediate and mesoscale ecosystem effects on population dynamics and biogeochemistry, virophages may have impacted their environment for millions of years by shaping the genomic landscape of their hosts through gene exchange and recombination. Typically, each virophage genome contains a few genes with homologs in eukaryotes, bacteria, GVs, or bacteriophages, indicating that virophages mediate horizontal gene transfer [2,18]. Combined with their ability to integrate into other genomes and their close relationship with eukaryotic Polintons, viruses of the family *Lavidaviridae* emerge as an influential group of mobile genetic elements in protozoa and microalgae.

## What distinguishes virophages from other host-parasite systems?

Hyperparasitism, or nested parasitism, is a widespread phenomenon in nature (see, e.g., [27]). Yet, virophages exhibit several idiosyncratic features that justify closer examination. Lavidaviruses are bone fide viruses in that they require a host cell for replication and transmit via an extracellular, capsid-enclosed state. However, whereas most other viruses depend on a host cell that is susceptible (i.e., allowing virus entry) and permissive (i.e., allowing virus replication), virophages split these requirements onto two different biological entities: they require a susceptible host cell and a permissive giant virus. Susceptibility is provided through specific receptor interactions between virophage capsid and cell surface (e.g., mavirus) or through phagocytosis of a virophage-GV composite (e.g., Sputnik). Permissiveness probably depends on whether the GV-encoded transcription machinery is compatible with the virophage genome. This situation emphasizes the concept of the virocell, in which the intracellular stage of the virus replication cycle is regarded as a viral organism, resulting in a cell that is controlled by the virus and thus behaves very differently from an uninfected cell [28]. The virocell status of GVs is particularly pronounced by the size and complexity of their virion factories, which provide enzymatic functions that are usually restricted to the nucleus. In that sense, virophages are parasites of the virocell. As with other host-parasite systems, the selective pressure put on GVs by virophages may eventually lead to the emergence of generic or specific resistance mechanisms against the virophage. Evidence for a specific defense mechanism comes from the *Acanthamoeba*–mimivirus–Zamilon system, in which the Zamilon virophage replicates only with mimiviruses of lineages B and C but not with those of lineage A [9]. Resistance in lineage A mimiviruses through the so-called MIMIVIRE system involves a multigene cluster encoding a Cas4 nuclease-like protein, but the specific mechanism of action is still unclear [29–31]. Another way for GVs to rid themselves of virophages is to prevent co-entry. Sputnik exhibits strong affinity for the glycosylated fibers of mimivirus capsids, and a fiberless mimivirus mutant was shown to be resistant to Sputnik [32]. Other defensive measures may include restriction-modification systems in GVs and mutations in GV-encoded transcription factors that support virophage gene expression. As an ultima ratio, GVs could give up their transcriptional independence altogether to escape virophage predation. This could have happened to viruses of the family *Phycodnaviridae*, most of which encode only a partial transcription system and require a nuclear phase for viral mRNA synthesis.

In conclusion, virophages are fascinating eukaryotic DNA viruses that have evolved to depend on a co-infecting GV instead of replicating in the host cell nucleus. They possess a high degree of genome mobility that may compensate for their unusual host requirements. The long co-evolution of virophages with their viral and cellular hosts has led to unique adaptations, such as the mutualistic *Cafeteria*–mavirus relationship, or the MIMIVIRE defense system in mimiviruses. Many more secrets will be revealed as we immerge deeper into the exciting microcosm of unicellular eukaryotes and their mobile genetic elements.
